# “Uropathogens and antimicrobial susceptibility patterns in urosepsis patients at kafr el sheikh University hospital: a cross-sectional study”

**DOI:** 10.1038/s41598-026-62193-z

**Published:** 2026-07-18

**Authors:** Ayat Shaban Mousa El Nahal, Ali Ibrahim, Elsayed Abdelhalim, Sally Aly Saleh Mohamed, Mo’men Mahmoud Saadoun, Moustafa Hamdy, Hebatallah Abdelmaksoud Abdelmonsef, Khaled Magdy Zeinelabden, Hossam Nabeeh, Diaa-Eldin Taha, Tarek Abdelbaky, Sally Hassan Essawy

**Affiliations:** 1 Medical Microbiology Department, Faculty of Medicine, Kafrelsheikh University, Kafrelsheikh, Egypt; 2 Urology Department, Faculty of Medicine, Kafrelsheikh University, Kafrelsheikh, Egypt; 3 Clinical Pathology Department, Faculty of Medicine, Kafrelsheikh University, Kafrelsheikh, Egypt; 4 Community Medicine Department, Faculty of Medicine, Kafrelsheikh University, Kafrelsheikh, Egypt

**Keywords:** Urosepsis, Antimicrobial susceptibility, Urinary tract infection, Blood culture, Diseases, Medical research, Microbiology, Urology

## Abstract

Urosepsis is a life-threatening consequence of urinary tract infections, often linked to obstruction, indwelling devices, and healthcare exposure. Increasing antimicrobial resistance among uropathogens complicates empirical therapy and adversely affects outcomes, making local surveillance essential for appropriate management. To determine the bacterial profile and antimicrobial susceptibility patterns of urosepsis cases admitted to Kafr El-Sheikh University Hospital. This cross-sectional study included 168 patients with confirmed urosepsis admitted to the Urology Unit between February 2024 and October 2025. Urosepsis was defined by identical organisms in urine and blood cultures with systemic sepsis and a SOFA score of ≥ 2. Cultures were processed using standard microbiological methods, with identification confirmed by biochemical testing and ATR-FTIR. Antimicrobial susceptibility testing was performed using the Kirby–Bauer method according to CLSI 2024 and verified by the BD Phoenix™ system. Multidrug-resistant (MDR) and ESBL-producing organisms were identified using standard criteria. The mean age of the patients was 56.2 ± 15.6 years, and 70.8% were male. Catheter-associated infections were identified in all cases (100%), reflecting the healthcare-associated nature of this cohort, while renal abscesses were detected in 26.2%. Gram-negative organisms predominated (90.5%), with *Escherichia coli* being the most frequently isolated pathogen. Statistical analysis revealed no significant demographic or clinical predictors of antimicrobial resistance patterns (*p* > 0.05). Gram-negative isolates showed higher susceptibility to carbapenems and fluoroquinolones. ESBL-producing isolates accounted for 24.34% and were susceptible to carbapenems. MDR isolates comprised 27.63% and showed high resistance to cephalosporins, piperacillin-tazobactam, and carbapenems. All gram-positive isolates were fully sensitive to vancomycin and teicoplanin. Catheter-associated urosepsis is mainly caused by resistant gram-negative pathogens. The high prevalence of ESBL and MDR strains underscores the need for early culture-guided therapy and continuous local antimicrobial surveillance to optimize outcomes.

**Supplementary Information:**

The online version contains supplementary material available at 10.1038/s41598-026-62193-z.

## Introduction

Urosepsis is a life-threatening organ dysfunction caused by a dysregulated host response to infection originating from the urinary tract and/or genital organs^1^. Urosepsis, in most cases, is caused by complicated urinary tract infections and accounts for 9–31% of all cases of septicemia globally. It affects approximately 7% to 12% of patients with nosocomial urinary tract infections and carries a fatality rate ranging from 15% to 40%, depending on the region and promptness of treatment^2–5^.

Urosepsis represents an escalating public health concern in Egypt. Its significance is driven by high rates of multidrug-resistant organisms, frequent hospital-acquired infections, and a heavy strain on the Egyptian intensive care unit system^6,7^. Local data indicate that urinary tract infections (UTIs) account for about 44% of microbial infections in Egyptian hospitals. About 24.5% of adult intensive care sepsis cases originate from the urogenital tract, with mortality rates often exceeding 40% in severe cases^8^.

The most common risk factor for urosepsis is obstruction to the free flow of urine, which may quickly lead to severe sepsis^1^. Other risk factors include old age, diabetes mellitus, immune suppression, nosocomial urinary tract infection acquired on a urology ward^,^ and prior urological interventions^9^. The severity and course of urosepsis depend on both the virulence of the pathogen and the patient’s specific immune response^2^.

Sources of pathogens causing urosepsis include infections of any genitourinary organ: the kidney (pyelonephritis, pyonephrosis, renal abscess), bladder (severe cystitis), prostate (acute bacterial prostatitis, post-transrectal ultrasound-guided prostate biopsy), testicular or scrotal (epididymo-orchitis, Fournier’s gangrene)^10^. The most common pathogens causing urinary tract infections (UTIs), and in turn urosepsis, are *Escherichia coli (E. coli)*, followed by *Proteus* species, *Enterobacter* species, *Klebsiella* species, *Pseudomonas aeruginosa (P. aeruginosa)*, and gram-positive bacteria^11^.

Bacterial endotoxins, such as lipopolysaccharide (LPS) from the cell wall of gram-negative organisms, appear to mediate the systemic manifestations of sepsis. These bacterial components activate the inflammatory, coagulation, and complement systems, stimulating the activity of monocytes, macrophages, neutrophils, and dendritic cells. In addition to stimulating inflammatory cells, endotoxin also directly binds to receptors in the endothelial cell membrane, which also promotes pro-inflammatory mediators^12^.

Diagnosis is initiated with a history, clinical assessment of systemic features, and urinary symptoms. Laboratory investigations should include a complete blood count, electrolytes and renal function tests, urinalysis, blood and urine cultures prior to antibiotic initiation, and sonographic examination of the urinary tract^13^. Positive blood cultures are the gold standard for diagnosing sepsis. Sepsis-related scoring systems such as the Sequential Organ Failure Assessment (SOFA) score and the Acute Physiology and Chronic Health Evaluation II (APACHE II) score can determine the severity of urosepsis and predict patient mortality^14^.

Urosepsis is a treatable condition. However, delayed management can lead to severe consequences, including renal failure, septic shock, and death^15^. High mortality is not only related to disease severity but also to delayed diagnosis and inadequate treatment of the disease^16^. Prompt therapy is essential, including antimicrobial treatment and eradication of the infection source, along with supportive measures for circulatory and respiratory stabilization, optimal management of the causative urinary tract disorder, and adjunctive therapies such as hemodialysis and glucocorticoid therapy^2^.

Antimicrobials are among the most important drugs in the management of patients with severe infections. Inappropriate use of antimicrobials can cause therapeutic failure in the individual patient and can contribute to the emergence of resistant pathogens, which might also readily spread in the hospital setting. Initial empirical treatment should provide effective broad antimicrobial coverage and should later be adapted to the culture results^9^.

Despite the significant public health burden and the escalating threat of antimicrobial resistance in Egypt, contemporary local epidemiological data, particularly concerning multidrug-resistant and carbapenem-resistant uropathogens in catheterized patients, remain scarce. Continuous local surveillance is crucial because resistance profiles vary significantly between different geographic regions and healthcare facilities. Therefore, this study aims to address this knowledge gap by determining the spectrum of bacteria causing urosepsis and their antimicrobial susceptibility patterns among admitted patients at our tertiary care center, thereby guiding evidence-based empirical treatment protocols.

## Materials and methods

### Study ethics and design

This cross-sectional study was conducted at the Urology Unit, Faculty of Medicine, Kafr Elsheikh University Hospital from February 2024 to October 2025. The study details and procedures were explained to all participants, and informed consent was obtained from all subjects and/or their legal guardians before enrollment. This study was approved by the Research Ethics Committee, Kafr Elsheikh University, before study initiation (approval number: KFSIRB200-226), with final approval dated 29/1/2024.

### Sampling and study participants

The sample size was calculated using the Epi-Info software statistical package. The criteria used for the calculation were as follows: 95% confidence level, 80% power of the study, 5% precision, and an expected cure rate for urosepsis of 88.3%, based on the results of a previous study^17^. The sample size based on the previously mentioned criteria was 160 participants.

The study was conducted on 168 patients with confirmed urosepsis admitted to the Urology Unit between February 2024 and October 2025. Urosepsis was defined as a urinary tract infection associated with systemic infection, confirmed by the isolation of identical bacterial pathogens from urine and blood cultures, together with clinical and laboratory evidence of organ dysfunction (SOFA score ≥ 2). Patients with positive blood cultures for microorganisms different from those identified in urine cultures and those with polymicrobial urine cultures were excluded. Furthermore, patients with concurrent infections at other anatomical sites, such as pneumonia or catheter-related bloodstream infections, were excluded from this study.

### Study procedure

All patients underwent complete history taking, with emphasis on urinary symptoms (dysuria, flank pain, frequency, and suprapubic discomfort) and relevant risk factors such as urinary catheterization, obstruction, urolithiasis, nephrostomy, double-J stent, or recent urological procedures. A full general examination was performed, focusing on temperature, blood pressure, heart rate, and mental status. Abdominal examination was performed with particular emphasis on assessment for costovertebral angle tenderness and suprapubic tenderness, in addition to abdominal palpation for detection of organomegaly. All participants underwent radiological and laboratory investigations, including abdominal ultrasonography and computed tomography (CT) to identify urinary obstruction, abscess formation, or hydronephrosis. Laboratory investigations included a complete blood count (CBC), serum C-reactive protein (CRP), serum creatinine, urine analysis, urine culture, and blood culture. The quick SOFA score was initially applied as a bedside screening tool for early identification of patients at risk of organ dysfunction and was considered positive in the presence of at least two of the following criteria: respiratory rate ≥ 22 breaths/min, altered mentation, or systolic blood pressure ≤ 100 mmHg. Sepsis-related organ dysfunction was subsequently confirmed by an increase in the SOFA score of ≥ 2 points^18^.

### Urine and blood culture procedures

Midstream clean-catch urine or catheterized urine samples were collected under aseptic conditions. Urine samples were inoculated using a semi-quantitative method on CLED agar (Cysteine Lactose Electrolyte Deficient) (Thermo Scientific™, C.L.E.D. Medium, USA, dehydrated). A sterile calibrated loop delivering 0.001 mL of urine was used to inoculate the culture medium, followed by incubation at 37 °C for 18–24 h. After incubation, visible colonies were counted, and the number of colony-forming units per milliliter (CFU/mL) was calculated by multiplying the colony count by 1000. Bacterial growth of ≥ 10^5 CFU/mL was interpreted as significant bacteriuria.

Blood samples drawn from patients were incubated in blood culture bottles (“Plus Aerobic/F Culture Vials”) and incubated at 35–37 °C for 5 days to monitor microbial growth using BD Bactec™ FX40 (BD Diagnostic Systems, Sparks, MD, USA)^19^. Blood culture bottles flagged as positive were subcultured on blood agar (Thermo Scientific™, USA, Blood Agar, dehydrated) and MacConkey’s agar (Thermo Scientific™, USA, MacConkey agar, dehydrated). All inoculated culture media were incubated at 35–37 °C for 24 h.

The preliminary identification of bacteria was performed through colony morphology, type of hemolysis on blood agar, and gram staining (Thermo Scientific™, USA, Gram Stain Kit). Identification of gram-positive and gram-negative bacteria was confirmed using a sequence of biochemical reactions; gram-positive bacteria were differentiated by catalase test, coagulase test, and bile esculin hydrolysis. Bile esculin agar was included to improve differentiation and confirmation of *Enterococcus* species, including *E. faecalis* and *E. faecium*, while gram-negative bacteria were identified using oxidase, indole, citrate, urease, triple sugar iron (TSI), and carbohydrate fermentation tests^20^. Further bacterial strain confirmation was performed using the ATR-FTIR device (Attenuated Total Reflectance–Fourier Transform Infrared spectroscopy, Italy)^21^.

Antimicrobial susceptibility testing was performed using the conventional Kirby–Bauer disk diffusion method. Antibiotics without disk diffusion breakpoints were tested using the gradient diffusion (E-test) method. All procedures were conducted according to the Clinical and Laboratory Standards Institute (CLSI, 2024) guidelines^22^. Briefly, bacterial suspensions were adjusted to 0.5 McFarland turbidity standard. The inoculum was then uniformly spread onto Mueller–Hinton agar (Thermo Scientific™, USA; Mueller Hinton Agar, dehydrated). Subsequently, selected antibiotic discs (Oxoid™, USA; antimicrobial susceptibility discs) were placed on the agar surface, and the plates were incubated at 35 °C for 16–24 h.

The antimicrobial disks used for gram-negative isolates included ampicillin (10 µg), cefuroxime (30 µg), cefotaxime (30 µg), ceftazidime (30 µg), ceftriaxone (30 µg), cefepime (30 µg), piperacillin/tazobactam (100/10 µg), gentamicin (10 µg), amikacin (30 µg), ciprofloxacin (5 µg), levofloxacin (5 µg), imipenem (10 µg), meropenem (10 µg), trimethoprim/sulfamethoxazole (TMP/SMX) (1.25/23.75 µg), and amoxicillin/clavulanate (20/10 µg). Colistin E-test strips (0.016–256 µg/mL) were used for determination of minimum inhibitory concentrations (MICs). Both ceftazidime/clavulanate (30/10 µg) and cefotaxime/clavulanate (30/10 µg) disks were used for phenotypic detection of extended-spectrum β-lactamase (ESBL) production. Certain antimicrobial agents were excluded for specific organisms according to their intrinsic resistance profiles and CLSI recommendations. The antibiotic disks used for *Enterococcus* species included high-level gentamicin (120 µg), ciprofloxacin (5 µg), ampicillin (10 µg), penicillin (10 units), vancomycin (30 µg), and teicoplanin (30 µg). For *Staphylococcus aureus* (*S. aureus*), the antibiotics tested included gentamicin (10 µg), ciprofloxacin (5 µg), TMP/SMX (1.25/23.75 µg), ampicillin (10 µg), cefuroxime (30 µg), penicillin (10 units), and cefoxitin (30 µg) as a surrogate marker for oxacillin resistance screening. Vancomycin and teicoplanin E-test strips (0.016–256 µg/mL) were used for determination of MICs according to the manufacturer’s instructions. Briefly, bacterial suspensions were adjusted to 0.5 McFarland turbidity and inoculated onto appropriate agar media. The antimicrobial strips were then applied, followed by incubation under standardized conditions. MIC values were determined at the point where the inhibition ellipse intersected the strip scale and were interpreted according to CLSI 2024 breakpoints.

Antimicrobial susceptibility results were interpreted according to CLSI 2024 guidelines using CLSI-recommended breakpoints for each organism^22^. Furthermore, the susceptibility results of drug-resistant organisms were cross-checked and confirmed by the BD Phoenix™ M50 (BD Diagnostic Systems, Sparks, MD, USA) automated identification and susceptibility system^23^.

Colistin susceptibility testing was initially performed using the E-test method for MIC determination, as disk diffusion is considered unsuitable for colistin evaluation due to its poor diffusion properties. Interpretation of colistin susceptibility results was based on CLSI 2024 breakpoints, which define only intermediate and resistant categories for colistin. To enhance the accuracy and reliability of colistin susceptibility assessment, especially among multidrug-resistant (MDR) and ESBL-producing isolates, results were further validated using the BD Phoenix™ M50 system, which utilizes a broth microdilution-based approach consistent with the CLSI reference method for colistin susceptibility testing.

Quality control (QC) testing was performed using standard reference strains recommended by CLSI to ensure the accuracy and reliability of antimicrobial susceptibility testing results. The QC strains included *E. coli* ATCC 25,922, *P. aeruginosa* ATCC 27,853, *A. baumannii* ATCC 19,606, *E. faecalis* ATCC 29,212, and *S. aureus* ATCC 25,923. QC strains were tested under the same conditions as clinical isolates, and results were accepted when they fell within CLSI-defined quality control ranges. These reference strains were also used to validate E-test and Phoenix-based MIC determinations, including colistin susceptibility testing, in accordance with CLSI 2024 and manufacturer recommendations.

ESBL production was detected according to CLSI 2024 guidelines using a two-step approach. Initial screening was performed using ceftazidime (30 µg), cefotaxime (30 µg), and ceftriaxone (30 µg) disks for *E. coli*, *Klebsiella pneumoniae (K. pneumoniae)*, and *K. oxytoca*, and ceftazidime (30 µg) or cefotaxime (30 µg) for *P. mirabilis*. Isolates showing inhibition zone diameters of ≤ 22 mm for ceftazidime, ≤ 27 mm for cefotaxime, or ≤ 25 mm for ceftriaxone were considered potential ESBL producers. Phenotypic confirmation was subsequently performed by combination disk diffusion testing using ceftazidime (30 µg) and ceftazidime–clavulanate (30/10 µg), as well as cefotaxime (30 µg) and cefotaxime–clavulanate (30/10 µg). An increase of ≥ 5 mm in inhibition zone diameter for either cephalosporin combined with clavulanate compared with the cephalosporin alone was interpreted as confirmation of ESBL production according to CLSI criteria.

MDR bacteria were detected as a lack of susceptibility to at least one antimicrobial agent across three or more categories^24^.

### Data management and analysis plan

The data were analyzed using the Statistical Package for the Social Sciences (SPSS version 25.0; IBM Corp., Armonk, NY, USA). The Kolmogorov–Smirnov test was used to assess the normality of quantitative data. Qualitative variables were presented using numbers and percentages. Numerical variables were expressed as median (IQR) or mean ± SD. Comparisons between groups were performed using the Monte Carlo exact test for categorical variables, and the Kruskal–Wallis test for continuous variables. A p-value < 0.05 was considered statistically significant.

## Results

This study included 168 patients diagnosed with urosepsis, with a mean age of 56.2 ± 15.6 years and **a** mean BMI of 24.4 ± 2.2 kg/m^2^. Male patients represented 70.8%, while female patients represented 29.2% of the total cohort. All patients had indwelling catheters. A history of surgical intervention was present in 109 patients (64.9%), while histories of DM and HTN were present in 28.0% and 36.3% of patients, respectively, and 12.5% of patients were stone formers. Renal and perinephric abscesses were present in 26.2% and 16.1% of the patients, respectively. A nephrostomy tube was present in 19.0% and a DJ stent was present in 14.9% of the patients **(**Table 1**).** Laboratory tests of the patients showed a median hemoglobin level of 12.6 g/dL, platelet count of 209 × 10⁹/L, white blood cell count of 14.5 × 10⁹/L, serum creatinine of 1.1 mg/dL, prothrombin concentration of 83%, and CRP of 191 mg/L **(**Table 2**).**

**Table 1:** Socio-demographic and clinical characteristics of the study participants (*N* = 168).

Studied variables		*N*	%
**Age (years) mean ± SD (range)**		56.2 ± 15.6 (7.0–82.0)	
**Sex**	**Female**	49	29.2%
	**Male**	119	70.8%
**BMI (kg/m** ^**2**^ **) mean ± SD**		24.4 ± 2.2	
**History of congenital anomalies**		10	6.0%
**Stone former**		21	12.5%
**History of surgical intervention**		109	64.9%
**Patients with indwelling catheters**		168	100%
**Presence of renal abscess**		44	26.2%
**Presence of perinephric abscess**		27	16.1%
**Presence of a DJ stent**		25	14.9%
**Presence of a nephrostomy tube**		32	19.0%
**History of DM**		47	28.0%
**History of HTN**		61	36.3%
**History of cardiac diseases**		12	7.1%
**History of hepatic diseases**		6	3.6%

BMI, body mass index; DJ, double-J; DM, diabetes mellitus; HTN, hypertension; SD, standard deviation.

**Table 2:** Laboratory characteristics of the study participants (*N* = 168).

Variable	Median	25th Percentile	75th Percentile
**Hemoglobin (Hb) g/dL**	12.6	11.0	14.5
**Platelet Count (×10⁹/L)**	209.0	179.5	241.0
**White Blood Cell Count (WBCs) (×10⁹/L)**	14.5	12.5	17.8
**Serum Creatinine (mg/dL)**	1.1	0.9	1.7
**Prothrombin Concentration (%)**	83.0	77.0	94.5
**CRP (mg/L)**	191.0	138.0	243.0

**Fig. 1:**
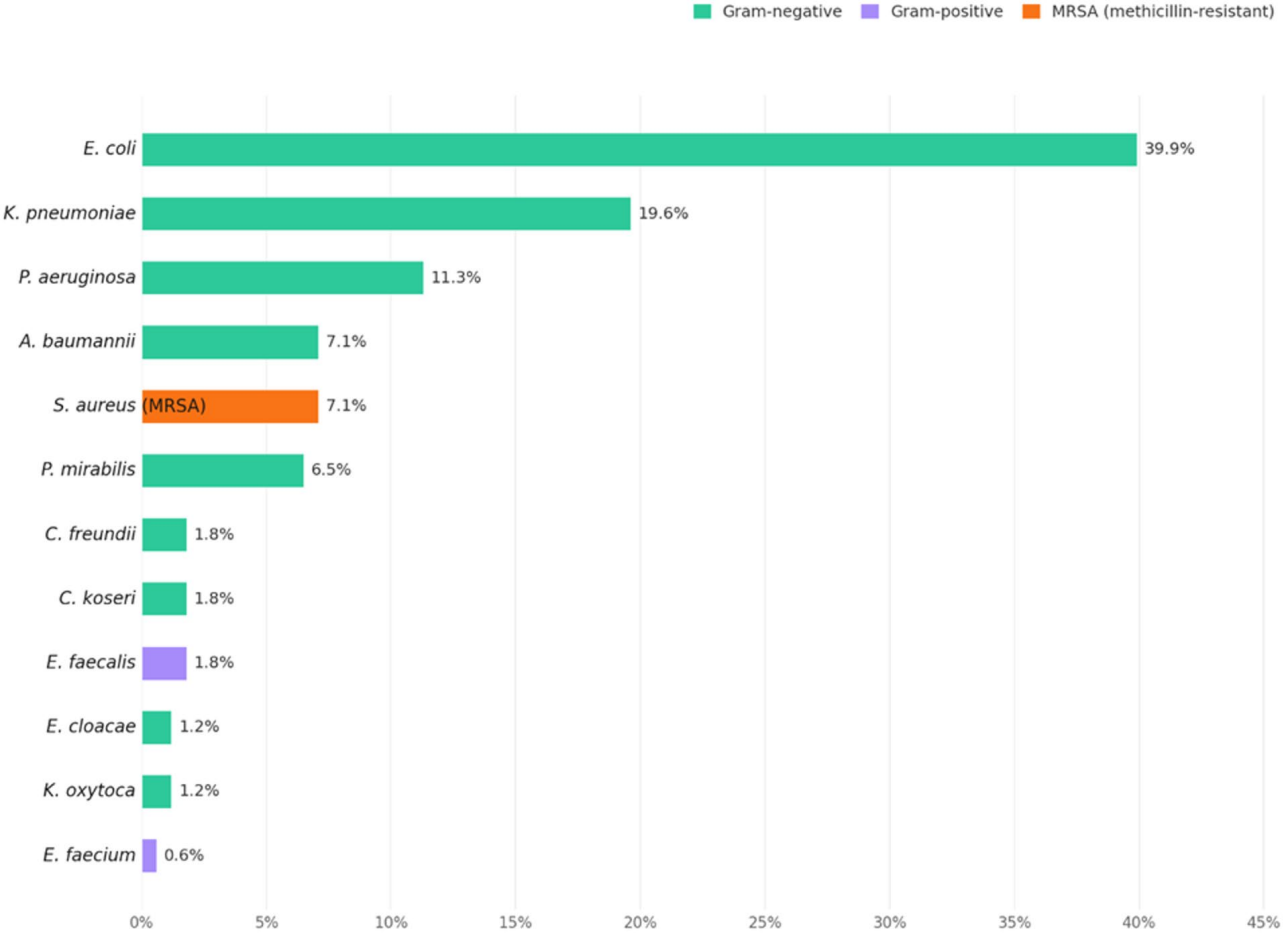
Percentage distribution of bacterial isolates by species. MRSA = methicillin-resistant Staphylococcus aureus.

**Fig. 2:**
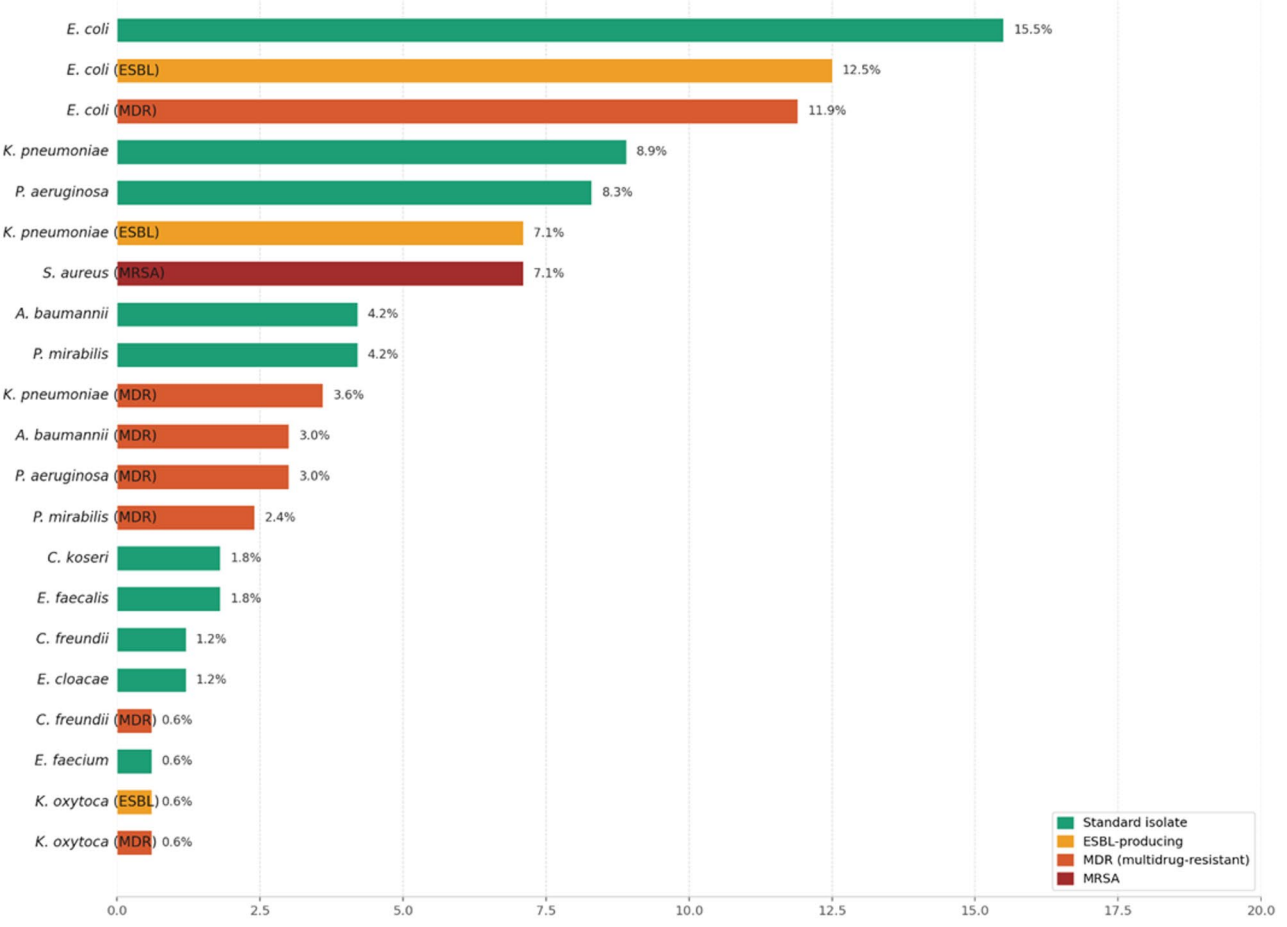
Percentage distribution of bacterial isolates by resistance Profile. MDR = multidrug-resistant; ESBL = extended-spectrum β-lactamase; MRSA = methicillin-resistant S. aureus.

A total of 168 bacterial isolates (16 species, 9 genera) were recovered, of which 152 (90.47%) were gram-negative (9 species) and 16 (9.53%) were gram-positive (3 species). *E. coli*,* K. pneumoniae*, and *P. aeruginosa* were the most prevalent gram-negative bacteria, accounting for 39.9%, 19.6%, and 11.3% of total isolates, respectively. *K. oxytoca* was the least frequently isolated gram-negative species (1.2%). Among gram-positive bacteria, methicillin-resistant *Staphylococcus aureus* (MRSA) was the most predominant (7.1%), whereas *Enterococcus faecium (E. faecium)* was the least frequently isolated (0.6%). ESBL-producing *E. coli*, MDR *E. coli*, and ESBL-producing *K. pneumoniae* were the most frequently detected resistant phenotypes (Figs. 1 and 2).

The antimicrobial susceptibility profile of gram-negative bacteria for the 16 antibacterial drugs tested is illustrated in Table 3. Ampicillin had the highest resistance rate (100%) among gram-negative bacteria, followed by cefuroxime (80.99%) and ceftazidime (77.63%). All gram-negative isolates other than *Proteus spp*. were sensitive to colistin. Gram-negative bacteria showed better sensitivity toward meropenem, imipenem, and amikacin, with sensitivity rates of 68.42%, 66.45%, and 65.10%, respectively.

**Table 3:** Antimicrobial susceptibility pattern of all gram-negative bacterial isolates among the study participants (*N* = 152).

Antibiotics	Total (*N*)	Sensitive		Intermediate		Resistant	
		*N*	%	*N*	%	*N*	%
**Gentamicin**	(*N* = 133)	84	63.2%	15	11.3%	34	25.6%
**Amikacin**	(*N* = 152)	99	65.1%	16	10.5%	37	24.3%
**Ciprofloxacin**	(*N* = 152)	62	40.8%	46	30.3%	44	28.9%
**Levofloxacin**	(*N* = 152)	61	40.1%	47	30.9%	44	28.9%
**TMP/SMX**	(*N* = 133)	77	57.9%	10	7.51%	46	34.58%
**Cefotaxime**	(*N* = 121)	25	20.7%	3	2.5%	93	76.9%
**Cefepime**	(*N* = 152)	56	36.8%	0	0.0%	96	63.2%
**Ampicillin**	(*N* = 121)	0	0.0%	0	0.0%	121	100.0%
**Piperacillin-tazobactam**	(*N* = 152)	70	46.0%	1	0.7%	81	53.3%
**Ceftazidime**	(*N* = 152)	30	19.70%	4	2.6%	118	77.6%
**Imipenem**	(*N* = 152)	101	66.4%	3	2.0%	48	31.6%
**Meropenem**	(*N* = 152)	104	68.4%	0	0.0%	48	31.6%
**Cefuroxime**	(*N* = 121)	15	12.4%	8	6.6%	98	81.0%
**Ceftriaxone**	(*N* = 121)	25	20.7%	3	2.5%	93	76.9%
**Amoxicillin-clavulanate**	(*N* = 121)	50	41.3%	4	3.3%	67	55.4%
**Colistin**	(*N* = 141)	141	100.0%	0	0.0%	0	0.0%

### TMP/SMX, trimethoprim/sulfamethoxazole

*E. coli*, the most frequently isolated bacterium, showed the lowest resistance rate (23.9%) to gentamicin, amikacin, levofloxacin, and ciprofloxacin, followed by meropenem and imipenem (29.9%). *K. pneumoniae*, the second most frequently isolated bacterium, showed the lowest resistance rate (18.2%) to meropenem and imipenem, followed by gentamicin and amikacin (21.2%). *P. aeruginosa*, the third most frequently isolated gram-negative bacterium, showed the lowest resistance rate (21.1%) to amikacin, followed by levofloxacin and ciprofloxacin (36.8%). *A. baumannii*, the fourth most frequently isolated bacterium, showed the lowest resistance rate (25.0%) to amikacin and TMP/SMX (Table 4).

**Table 4:** Antimicrobial resistance pattern of each gram-negative bacterial isolate.

Antibiotic	Pseudomonas aeruginosa (*N* = 19) *R* *n* (%)	Proteus mirabilis (*N* = 11) *R* *n* (%)	Klebsiella pneumoniae (*N* = 33) *R* *n* (%)	Klebsiella oxytoca (*N* = 2) *R* *n* (%)	Enterobacter cloacae (*N* = 2) *R* *n* (%)	Escherichia coli (*N* = 67)	Citrobacter koseri (*N* = 3) *R* *n* (%)	Citrobacter freundii (*N* = 3) *R* *n* (%)	Acinetobacter baumannii (*N* = 12) *R* *n* (%)
Gentamicin	NA	4(36.4%)	7(21.2%)	1(50.0%)	2(100.0%)	16(23.9%)	0(0.0%)	0(0.0%)	4(33.3%)
Amikacin	4(21.1%)	4(36.4%)	7(21.2%)	1(50.0%)	2(100.0%)	16(23.9%)	0(0.0%)	0(0.0%)	3(25.0%)
**Ciprofloxacin**	**7(36.8%)**	**2(18.2%)**	**11(33.3%)**	**1(50.0%)**	**0(0.0%)**	**16(23.9%)**	**0(0.0%)**	**1(33.3%)**	**6(50.0%)**
**Levofloxacin**	**7(36.8%)**	**2(18.2%)**	**11(33.3%)**	**1(50.0%)**	**0(0.0%)**	**16(23.9%)**	**0(0.0%)**	**1(33.3%)**	**6(50.0%)**
**TMP/SMX**	**NA**	**4(36.4%)**	**14(42.4%)**	**2(100.0%)**	**0(0.0%)**	**22(32.8%)**	**0(0.0%)**	**1(33.3%)**	**3(25.0%)**
**Cefotaxime**	**NA**	**11(100.0%)**	**25(75.7%)**	**2(100.0%)**	**2(100.0%)**	**48(71.6%)**	**3(100.0%)**	**2(66.7%)**	**NA**
**Cefepime**	**11(57.9%)**	**10(90.9%)**	**21(63.6%)**	**2(100.0%)**	**2(100.0%)**	**41(61.2%)**	**0(0.0%)**	**1(33.3%)**	**8(66.7%)**
**Ampicillin**	**NA**	**11(100.0%)**	**33(100.0%)**	**2(100.0%)**	**2(100.0%)**	**67(100.0%)**	**3(100.0%)**	**3(100.0%)**	**NA**
**Piperacillin-tazobactam**	**10(52.6%)**	**8(72.7%)**	**17(51.5%)**	**2(100.0%)**	**2(100.0%)**	**33(49.3%)**	**0(0.0%)**	**2(66.7%)**	**7(58.3%)**
**Ceftazidime**	**14(73.7%)**	**11(100.0%)**	**25(75.7%)**	**2(100.0%)**	**2(100.0%)**	**48(71.6%)**	**3(100.0%)**	**2(66.7%)**	**11(91.7%)**
**Imipenem**	**9(47.4%)**	**4(36.4%)**	**6(18.2%)**	**1(50.0%)**	**0(0.0%)**	**20(29.9%)**	**0(0.0%)**	**1(33.3%)**	**7(58.3%)**
**Meropenem**	**9(47.4%)**	**4(36.4%)**	**6(18.2%)**	**1(50.0%)**	**0(0.0%)**	**20(29.9%)**	**0(0.0%)**	**1(33.3%)**	**7(58.3%)**
**Cefuroxime**	**NA**	**11(100.0%)**	**27(81.8%)**	**2(100.0%)**	**2(100.0%)**	**50(74.6%)**	**3(100.0%)**	**3(100.0%)**	**NA**
**Ceftriaxone**	**NA**	**11(100.0%)**	**25(75.7%)**	**2(100.0%)**	**2(100.0%)**	**48(71.6%)**	**3(100.0%)**	**2(66.7%)**	**NA**
**Amoxicillin-clavulanate**	**NA**	**9(81.8%)**	**15(45.4%)**	**2(100.0%)**	**2(100.0%)**	**33(49.3%)**	**3(100.0%)**	**3(100.0%)**	**NA**
**Colistin**	**0(0.0%)**	**NA**	**0(0.0%)**	**0(0.0%)**	**0(0.0%)**	**0(0.0%)**	**0(0.0%)**	**0(0.0%)**	**0(0.0%)**

TMP/SMX, trimethoprim/sulfamethoxazole; R, resistant; NA, not applicable. The intermediate group was included in the sensitive group.

Among 152 gram-negative isolates, ESBL production was detected in 37 (24.3%) isolates, all of which were susceptible to carbapenems. Meanwhile, MDR was observed in 42 (27.6%) isolates, which demonstrated resistance to multiple β-lactams and carbapenems, but remained susceptible to colistin (Table 5).

**Table 5:** Antibiotic susceptibility profile of resistant bacterial isolates among the study participants.

Antibiotics	Total (*N*)	Resistant *n* (%)
MRSA (*N* = 12)		
Gentamicin	(*N* = 12)	4 (33.3%)
**Ciprofloxacin**	**(N = 12)**	**4 (33.3%)**
**TMP/SMX**	**(N = 12)**	**4 (33.3%)**
**Cefuroxime**	**(N = 12)**	**12 (100.0%)**
**Oxacillin**	**(N = 12)**	**12 (100.0%)**
**Penicillin**	**(N = 12)**	**12 (100.0%)**
**Vancomycin**	**(N = 12)**	**0 (0.0%)**
**Teicoplanin**	**(N = 12)**	**0 (0.0%)**
**ESBL (N = 37)**		
**Gentamicin**	**(N = 37)**	**5 (13.5%)**
**Amikacin**	**(N = 37)**	**5 (13.5%)**
**Ciprofloxacin**	**(N = 37)**	**8 (21.6%)**
**Levofloxacin**	**(N = 37)**	**8 (21.6%)**
**TMP/SMX**	**(N = 37)**	**13 (35.1%)**
**Cefotaxime**	**(N = 37)**	**37 (100.0%)**
**Cefepime**	**(N = 37)**	**37 (100.0%)**
**Ampicillin**	**(N = 37)**	**37 (100.0%)**
**Piperacillin-tazobactam**	**(N = 37)**	**24 (64.9%)**
**Ceftazidime**	**(N = 37)**	**37 (100.0%)**
**Imipenem**	**(N = 37)**	**0 (0.0%)**
**Meropenem**	**(N = 37)**	**0 (0.0%)**
**Cefuroxime**	**(N = 37)**	**37 (100.0%)**
**Ceftriaxone**	**(N = 37)**	**37 (100.0%)**
**Amoxicillin-clavulanate**	**(N = 37)**	**17 (45.9%)**
**Colistin**	**(N = 37)**	**0 (0.0%)**
**MDR (N = 42)**		
**Gentamicin**	**(N = 37)**	**23 (62.2%)**
**Amikacin**	**(N = 42)**	**27 (64.3%)**
**Ciprofloxacin**	**(N = 42)**	**30 (71.4%)**
**Levofloxacin**	**(N = 42)**	**30 (71.4%)**
**TMP/SMX**	**(N = 37)**	**23 (62.2%)**
**Cefotaxime**	**(N = 31)**	**31 (100.0%)**
**Cefepime**	**(N = 42)**	**42 (100.0%)**
**Ampicillin**	**(N = 31)**	**31 (100.0%)**
**Piperacillin-tazobactam**	**(N = 42)**	**42 (100.0%)**
**Ceftazidime**	**(N = 42)**	**42 (100.0%)**
**Imipenem**	**(N = 42)**	**42 (100.0%)**
**Meropenem**	**(N = 42)**	**42 (100.0%)**
**Cefuroxime**	**(N = 31)**	**31 (100.0%)**
**Ceftriaxone**	**(N = 31)**	**31 (100.0%)**
**Amoxicillin-clavulanate**	**(N = 31)**	**31 (100.0%)**
**Colistin**	**(N = 38)**	**0 (0.0%)**

MRSA, methicillin-resistant Staphylococcus aureus; ESBL, extended-spectrum beta-lactamase-producing organisms; MDR, multidrug-resistant organisms; TMP/SMX, trimethoprim/sulfamethoxazole. The intermediate group was included in the sensitive group.

Table 6 demonstrates the antimicrobial resistance pattern of gram-positive bacteria for nine antibacterial drugs. All gram-positive isolates were sensitive to vancomycin and teicoplanin (100%).

MRSA, the most frequently isolated gram-positive bacterium, showed the lowest resistance rate to TMP/SMX, ciprofloxacin, and gentamicin (33.3%). *Enterococcus faecalis*, the second most frequently isolated gram-positive bacterium, showed intermediate susceptibility to ciprofloxacin in all isolates. The lowest resistance rate (33.3%) was observed for high-level gentamicin, ampicillin, and penicillin. *Enterococcus faecium*, which was the least prevalent gram-positive isolate, exhibited 100% susceptibility to high-level gentamicin, ciprofloxacin, ampicillin, and penicillin (Table 7). Regarding the association between clinical characteristics and antimicrobial resistance patterns among the 168 urosepsis patients, no clinical or demographic characteristics were found to be statistically significant predictors of antimicrobial resistance in this cohort (Table 8).

**Table 6:** Antimicrobial resistance pattern of all gram-positive bacterial isolates among the study participants (*N* = 16).

Antibiotics	Total (*N*)	Resistant *n* (%)
Gentamicin	(*N* = 16)	5 (31.2%)
Ciprofloxacin	(*N* = 16)	4 (25.0%)
**TMP/SMX**	**(N = 12)**	**4 (33.3%)**
**Cefuroxime**	**(N = 12)**	**12 (100.0%)**
**Oxacillin**	**(N = 12)**	**12 (100.0%)**
**Vancomycin**	**(N = 16)**	**0 (0.0%)**
**Penicillin**	**(N = 16)**	**13 (81.2%)**
**Ampicillin**	**(N = 4)**	**1 (25.0%)**
**Teicoplanin**	**(N = 16)**	**0 (0.0%)**

TMP/SMX, trimethoprim/sulfamethoxazole. The intermediate group was included in the sensitive group.

**Table 7:** Antimicrobial resistance pattern of each gram-positive bacterial isolate.

Antibiotics	*MRSA (N = 12)* *R* N(%)	*Enterococcus faecalis (N = 3)* *R* N (%)	*Enterococcus faecium (N = 1)* *R* *n* (%)
Gentamicin	4 (33.3%)	1 (33.3%)	0 (0.0%)
Ciprofloxacin	4 (33.3%)	0 (0.0%)	0 (0.0%)
**TMP/SMX**	**4 (33.3%)**	**NA**	**NA**
**Ampicillin**	**NA**	**1 (33.3%)**	**0 (0.0%)**
**Cefuroxime**	**12 (100.0%)**	**NA**	**NA**
**Oxacillin**	**12 (100.0%)**	**NA**	**NA**
**Penicillin**	**12 (100.0%)**	**1 (33.3%)**	**0 (0.0%)**
**Vancomycin**	**0 (0.0%)**	**0 (0.0%)**	**0 (0.0%)**
**Teicoplanin**	**0 (0.0%)**	**0 (0.0%)**	**0 (0.0%)**

MRSA, methicillin-resistant Staphylococcus aureus; TMP/SMX, trimethoprim/sulfamethoxazole; NA, not applicable; R, resistant. The intermediate group was included in the sensitive group.

**Table 8:** Association between clinical characteristics and antimicrobial resistance patterns among patients with urosepsis (*N* = 168).

Studied variables		Negative (*N* = 77)		ESBL (*N* = 37)		MDR (*N* = 42)		MRSA (*N* = 12)		*P*-value
		*N*	%	*N*	%	*N*	%	*N*	%	
**Age (years) mean ± SD**		56.6 ± 14.2		54.2 ± 16.7		59.0 ± 16.2		50.2 ± 18.1		0.344
**Sex**	**Female**	20	40.8%	11	22.4%	13	26.5%	5	10.2%	0.729
	**Male**	57	47.9%	26	21.8%	29	24.4%	7	5.9%	
**BMI (kg/m** ^**2**^ **)**		24.6 ± 2.4		24.6 ± 1.6		24.1 ± 2.1		23.6 ± 2.1		0.274
**History of congenital anomalies**		4	40.0%	4	40.0%	2	20.0%	0	0.0%	0.490
**Stone former**		11	52.4%	5	23.8%	3	14.3%	2	9.5%	0.697
**History of surgical intervention**		53	48.6%	24	22.0%	25	22.9%	7	6.4%	0.747
**CAUTI**		76	45.8%	37	22.3%	41	24.7%	12	7.2%	> 0.999
**Presence of renal abscess**		19	43.2%	13	29.5%	9	20.5%	3	6.8%	0.559
**Presence of perinephric abscess**		12	44.4%	5	18.5%	9	33.3%	1	3.7%	0.639
**Presence of a DJ stent**		8	32.0%	10	40.0%	6	24.0%	1	4.0%	0.108
**Presence of a nephrostomy tube**		19	59.4%	3	9.4%	6	18.8%	4	12.5%	0.076
**History of DM**		23	48.9%	10	21.3%	10	21.3%	4	8.5%	0.878
**History of HTN**		31	50.8%	13	21.3%	14	23.0%	3	4.9%	0.710
**History of cardiac diseases**		8	66.7%	3	25.0%	1	8.3%	0	0.0%	0.266
**History of hepatic diseases**		3	50.0%	1	16.7%	1	16.7%	1	16.7%	0.848
**History of renal diseases**		6	50.0%	2	16.7%	2	16.7%	2	16.7%	0.544

ESBL, extended-spectrum β-lactamase; MDR, multidrug-resistant; MRSA, methicillin-resistant Staphylococcus aureus; BMI, body mass index; CAUTI, catheter-associated urinary tract infection; DJ, double-J; DM, diabetes mellitus; HTN, hypertension; SD, standard deviation.

## Discussion

This study provides a comprehensive analysis of the microbial epidemiology and antimicrobial resistance patterns in patients admitted with urosepsis at a tertiary care university hospital. Our findings reveal a highly challenging epidemiological profile: a predominance of MDR gram-negative pathogens in catheterized patients, coupled with a substantial reduction in carbapenem susceptibility among MDR isolates, which limits their reliability as empirical therapeutic options in this subgroup. These findings highlight the need for strict antimicrobial stewardship and the judicious use of last-resort agents such as colistin.

In contrast to uncomplicated urinary tract infections (UTIs), which predominantly affect females, our cohort exhibited a male predominance (70.8%). This demographic shift is characteristic of complicated urosepsis and is directly attributable to the high prevalence of obstructive uropathy and urological interventions in our male population. The study population was characterized by advanced age and major comorbidities, including hypertension and diabetes mellitus, creating an immunocompromised milieu susceptible to systemic invasion^25^. Clinically, the severity of sepsis in our cohort was evidenced by markedly elevated inflammatory markers (median CRP 191 mg/L) and severe structural complications such as renal and perinephric abscesses in over 40% of patients. This underscores that urosepsis in our setting is not merely an infection but a complex interplay between host factors, obstruction, and virulent pathogens that requires immediate source control alongside antimicrobial therapy^26^. Notably, our inferential statistical analysis revealed that none of these individual clinical or demographic characteristics were statistically significant predictors of specific antimicrobial resistance patterns (*p* > 0.05, Table 8). This crucial finding implies that the acquisition of ESBL or MDR pathogens in our cohort is universally driven by the shared environmental risk of prolonged catheterization and nosocomial exposure, rather than specific patient-level comorbidities.

Consistent with global and regional surveillance data for complicated UTIs, gram-negative bacteria accounted for the vast majority (90.5%) of isolates. While *E. coli* remained the leading pathogen (39.9%), the prevalence of *K. pneumoniae* (19.6%) was notably high. This distribution mirrors recent findings from other Egyptian tertiary centers, which report a similar shift toward *Klebsiella* species in hospital-acquired infections^27^. This pattern is strongly linked to the universal use of indwelling urinary catheters in our cohort (100%), as catheter-associated urosepsis creates a biofilm-mediated environment that selects for resistant *Enterobacteriaceae*, distinguishing it from community-acquired infections often driven by more susceptible strains^28^.

A notable finding in our study is the substantial burden of non-fermenting gram-negative bacilli, particularly *P. aeruginosa and A. baumannii*. Unlike *Enterobacteriaceae*, these pathogens possess intrinsic resistance mechanisms and a high capacity for biofilm formation on urological devices. Our *A. baumannii* isolates showed extensive resistance to third- and fourth-generation cephalosporins (Table 4), consistent with the “critical priority” status assigned to this pathogen by the WHO and with recent Egyptian genomic studies highlighting the dissemination of MDR clones in intensive care and urology units^29^. The persistence of these pathogens underscores the need for rigorous infection control measures during catheter manipulation to prevent outbreaks.

The most concerning finding of this study is the severity of antimicrobial resistance among the identified MDR isolates. While 24.3% of gram-negative isolates were ESBL producers, the distinct subset of MDR isolates (27.6%) demonstrated an extensively resistant profile. Notably, all MDR isolates in our cohort exhibited phenotypic non-susceptibility to both imipenem and meropenem, suggestive of the local emergence of Carbapenem-Resistant Enterobacteriaceae (CRE). This pattern is consistent with reports of carbapenemase-mediated resistance increasingly described in Egypt, including blaNDM and blaOXA-48^30,31^. However, confirmation of specific resistance mechanisms requires molecular characterization, which was not performed in this study. These findings highlight a substantial challenge to carbapenem efficacy in our locality and underscore the importance of local resistance surveillance^32^.

The observed resistance profiles pose a substantial challenge to standard empiric therapy guidelines. First-line agents such as third-generation cephalosporins showed resistance rates that render them unsuitable for empiric use in this cohort (Table 3). Notably, while MDR isolates exhibited high phenotypic resistance to carbapenems, they retained in vitro susceptibility to colistin. However, we strongly emphasize that these findings must not encourage its empirical use, especially given recent national data documenting emerging colistin resistance among gram-negative isolates in Egypt^33^. Therefore, colistin must remain strictly regulated as a last-resort agent, restricted exclusively to culture-proven, carbapenem-resistant infections to prevent the rapid emergence of pan-drug-resistant superbugs.

Interestingly, our findings revealed that resistance to trimethoprim/sulfamethoxazole (co-trimoxazole) was comparatively moderate (42.8% among gram-negative and 33.3% among gram-positive isolates) when contrasted with the observed very high resistance rates for third-generation cephalosporins. This relatively preserved activity may be attributed to the reduced reliance on co-trimoxazole in recent empirical treatment protocols. The decreased prescription of this older antimicrobial agent has likely relieved the selective pressure on uropathogens, suggesting a potential, albeit limited, role for co-trimoxazole as a targeted step-down therapy once susceptibility is confirmed. Among the gram-positive isolates, MRSA was the most frequently identified organism and demonstrated resistance to all tested beta-lactams (Table 5). Importantly, all gram-positive isolates retained full susceptibility to vancomycin and teicoplanin. Given the relatively low overall prevalence of MRSA (7.1%), the routine empirical use of anti-MRSA agents is not justified. However, in patients with indwelling devices who clinically deteriorate despite adequate gram-negative coverage, or those with known prior MRSA colonization, the targeted addition of anti-MRSA agents may be considered to mitigate the risk of metastatic infection^34^.

## Conclusion

In conclusion, catheter-associated urosepsis in our tertiary care setting is dominated by gram-negative pathogens with high rates of multidrug and carbapenem resistance. The high phenotypic resistance of MDR isolates to carbapenems and their retained susceptibility to colistin highlight a critical challenge to current empirical treatment strategies. These findings mandate immediate revision of local empiric treatment protocols, emphasizing strict antibiotic stewardship and the urgent need for rapid diagnostic tools to guide early targeted therapy^34,35^.

### Strengths and limitations of the study

Strengths of this study include the strict microbiological inclusion criteria requiring concordant urine and blood culture isolates, the stratification of results into ESBL and MDR phenotypes, and the confirmation of antimicrobial susceptibility results using an automated platform (BD Phoenix™) in addition to standard disk diffusion.

However, this study has several limitations. **First**, the single-center design may restrict generalizability to other geographic regions or healthcare settings. **Second**, the inclusion of a cohort consisting entirely of catheterized patients means our findings specifically reflect catheter-associated urosepsis and cannot be directly generalized to non-catheterized or community-acquired urosepsis cases. **Third**, while phenotypic resistance was well-characterized, molecular genotyping to identify specific resistance genes (such as carbapenemase or mcr genes) was not performed, representing an avenue for future research. **Fourth**, although colistin MIC results for drug-resistant isolates (MDR and ESBL-producing organisms) were cross-validated using the BD Phoenix™ broth microdilution-based system, broth microdilution was not applied as the primary reference method across the entire isolate collection. E-test results for the remaining isolates were not independently confirmed by broth microdilution, representing a residual methodological limitation given documented discordance between gradient diffusion and broth microdilution for colistin testing. **Finally**, this study primarily evaluated in vitro antimicrobial susceptibility profiles without longitudinally assessing patient clinical outcomes, such as treatment failure, length of hospital stay, or mortality rates. Future prospective studies are needed to directly correlate these in vitro resistance patterns with clinical prognosis.

### Ethical approval

All procedures performed in this study were in accordance with the ethical standards of the institutional research committee and with the 1964 Declaration of Helsinki and its later amendments.

## Supplementary Information

Below is the link to the electronic supplementary material.

Supplementary Material 1

Supplementary Material 2

Supplementary Material 3

Supplementary Material 4

Supplementary Material 5

## Data Availability

The data is contained within the manuscript, any missing details will be available from the corresponding author on reasonable request.

## Abbreviations

MDR: multidrug-resistant
MRSA: Methicillin-Resistant Staphylococcus aureus
UTIs: urinary tract infections
ESBL: extended-spectrum beta-lactamase
CRE: Carbapenem-Resistant Enterobacteriaceae
MRSA: Methicillin-Resistant Staphylococcus aureus
PCR: polymerase chain reaction
CRP: C-reactive protein
