# Supplementary material for: “Uropathogens and antimicrobial susceptibility patterns in urosepsis patients at kafr el sheikh University hospital: a cross-sectional study”
Source: Sci Rep. 2026 Jul 18;16:22544. doi: 10.1038/s41598-026-62193-z (PMC13380607; doi:10.1038/s41598-026-62193-z)
Supplement: Supplementary file 1 — Supplementary Material 1 [file 41598_2026_62193_MOESM1_ESM.docx]

# Clinical and Laboratory Standards Institute Disk Diffusion Zone Diameter Breakpoints For Enterobacterales

| Antimicrobial Agent | Disk Content (µg) | Interpretative categories and zone diameter breakpoints, nearest whole mm | | | |
| --- | --- | --- | --- | --- | --- |
|  |  | S | SDD | I | R |
| Ampicillin | 10 | ≥17 | - | 14–16 | /≤13 |
| Cefuroxime | 30 | ≥23 | - | 15–22 | ≤14 |
| Cefotaxime | 30 | ≥26 | - | 23–25 | ≤22 |
| Ceftazidime | 30 | ≥21 | - | 18–20 | ≤17 |
| Ceftriaxone | 30 | ≥23 | - | 20–22 | ≤19 |
| Cefepime | 30 | ≥25 | 19–24 | - | ≤18 |
| Piperacillin/Tazobactam | 100/10 | ≥25 | 21-24 | - | ≤20 |
| Gentamicin | 10 | ≥18 |  | 15-17 | ≤14 |
| Amikacin | 30 | ≥20 | - | 17-19 | ≤16 |
| Ciprofloxacin | 5 | ≥26 | - | 22-25 | ≤21 |
| Levofloxacin | 5 | ≥21 | - | 17-20 | ≤16 |
| Imipenem | 10 | ≥23 | - | 20–22 | ≤19 |
| Meropenem | 10 | ≥23 | - | 20–22 | ≤19 |
| Trimethoprim/Sulfamethoxazole | 1.25/23.75 | ≥16 | - | 11-15 | ≤10 |
| Amoxicillin/Clavulanate | 20/10 | ≥18 | - | 14–17 | ≤13 |

N.B: S = Sensitive, I = Intermediate, R = Resistant, SDD=Susceptible –Dose –Dependent

**Clinical and Laboratory Standards Institute Disk Diffusion Zone Diameter Breakpoints For *Acinetobacter baumannii***

| Antimicrobial Agent | Disk Content (µg) | Interpretative categories and zone diameter breakpoints, nearest whole mm | | |
| --- | --- | --- | --- | --- |
|  |  | S | I | R |
| Ceftazidime | 30 | ≥18 | 15–17 | ≤14 |
| Cefepime | 30 | ≥18 | 15–17 | ≤14 |
| Piperacillin/Tazobactam | 100/10 | ≥21 | 18-20 | ≤17 |
| Gentamicin | 10 | ≥19 | 14-18 | ≤13 |
| Amikacin | 30 | ≥20 | 17-19 | ≤16 |
| Ciprofloxacin | 5 | ≥21 | 16-20 | ≤15 |
| Levofloxacin | 5 | ≥17 | 14-16 | ≤13 |
| Imipenem | 10 | ≥23 | 20–22 | ≤19 |
| Meropenem | 10 | ≥23 | 20–22 | ≤19 |
| Trimethoprim/Sulfamethoxazole | 1.25/23.75 | ≥16 | 11-15 | ≤10 |

N.B: S = Sensitive, I = Intermediate, R = Resistant

**Clinical and Laboratory Standards Institute Disk Diffusion Zone Diameter Breakpoints For** ***Pseudomonas aeruginosa***

| Antimicrobial Agent | Disk Content (µg) | Interpretative categories and zone diameter breakpoints, nearest whole mm | | |
| --- | --- | --- | --- | --- |
|  |  | S | I | R |
| Ceftazidime | 30 | ≥18 | 15–17 | ≤14 |
| Cefepime | 30 | ≥18 | 15–17 | ≤14 |
| Piperacillin/Tazobactam | 100/10 | ≥22 | 18-21 | ≤17 |
| Amikacin | 30 | ≥17 | 15-16 | ≤14 |
| Ciprofloxacin | 5 | ≥25 | 19-24 | ≤18 |
| Levofloxacin | 5 | ≥22 | 15-21 | ≤14 |
| Imipenem | 10 | ≥19 | 16–18 | ≤15 |
| Meropenem | 10 | ≥19 | 16–18 | ≤15 |

N.B: S = Sensitive, I = Intermediate, R = Resistant

**Clinical and Laboratory Standards Institute Disk Diffusion Zone Diameter Breakpoints For** ***Staphylococcus aureus***

| Antimicrobial Agent | Disk Content (µg) | Interpretative categories and zone diameter breakpoints, nearest whole mm | | |
| --- | --- | --- | --- | --- |
|  |  | S | I | R |
| Gentamicin | 10 | ≥15 | 13–14 | ≤12 |
| Ciprofloxacin | 5 | ≥21 | 16-20 | ≤15 |
| Trimethoprim/Sulfamethoxazole | 1.25/23.75 | ≥16 | 11-15 | ≤10 |
| Ampicillin | 10 | ≥25 | 19-24 | ≤18 |
| Cefuroxime | 30 | ≥22 | 15-21 | ≤14 |
| Penicillin | 10 units | ≥29 | - | ≤28 |
| Cefoxitin | 30 | ≥22 | - | ≤21 |

N.B:S = Sensitive, I = Intermediate, R = Resistant, Cefoxitin surrogate test for oxacillin screening

**Clinical and Laboratory Standards Institute Disk Diffusion Zone Diameter Breakpoints For *Enterococcus* spescies.**

| Antimicrobial Agent | Disk Content (µg) | Interpretative categories and zone diameter breakpoints, nearest whole mm | | |
| --- | --- | --- | --- | --- |
|  |  | S | I | R |
| Penicillin | 10 units | ≥15 | - | ≤14 |
| Ampicillin | 10 | ≥17 | - | ≤16 |
| Ciprofloxacin | 5 | ≥21 | 16-20 | ≤15 |
| High-level Gentamicin | 120 | ≥10 | 7-9 (inco nclusive) | 6 |
| Vancomycin | 30 | ≥17 | 15-16 | ≤14 |
| Teicoplanin | 30 | ≥14 | 11-13 | ≤10 |

N.B:S = Sensitive, I = Intermediate, R = Resistant

**Footnotes:**

- For gram negative bacteria ,The E-test was used to determine the minimum inhibitory concentration (MIC) of colistin. MIC breakpoints and interpretive categories were defined as follows: intermediate ≤2 µg/mL and resistant ≥4 µg/mL.
- For *Staphylococcus aureus* ,The E-test was used to determine the MICs of vancomycin and teicoplanin. MIC breakpoints and interpretive categories were defined as follows: for vancomycin, susceptible ≤2 µg/mL, intermediate 4–8 µg/mL, and resistant ≥16 µg/mL; for teicoplanin, susceptible ≤8 µg/mL, intermediate 16 µg/mL, and resistant ≥32 µg/mL.


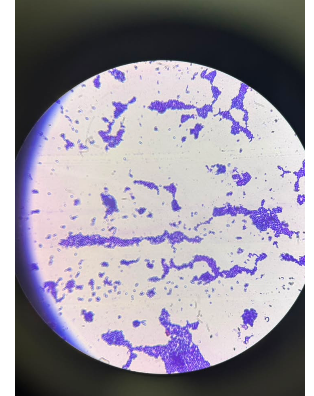


Figure 1:Gram positive cocci in clusters


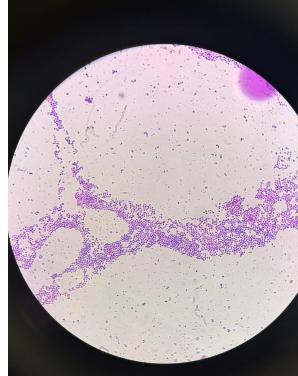


Figure 2: Gram negative coccobacilli


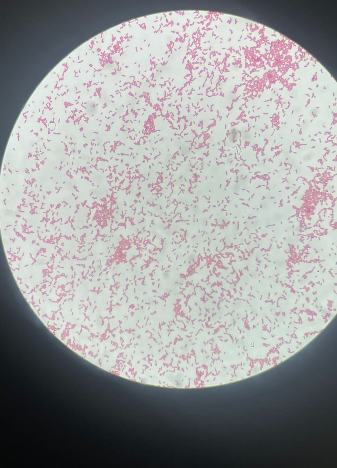


Figure 3:Gram negative bacilli
