# Supplementary figures and images for: “Uropathogens and antimicrobial susceptibility patterns in urosepsis patients at kafr el sheikh University hospital: a cross-sectional study”
Source: Sci Rep. 2026 Jul 18;16:22544. doi: 10.1038/s41598-026-62193-z (PMC13380607; doi:10.1038/s41598-026-62193-z)

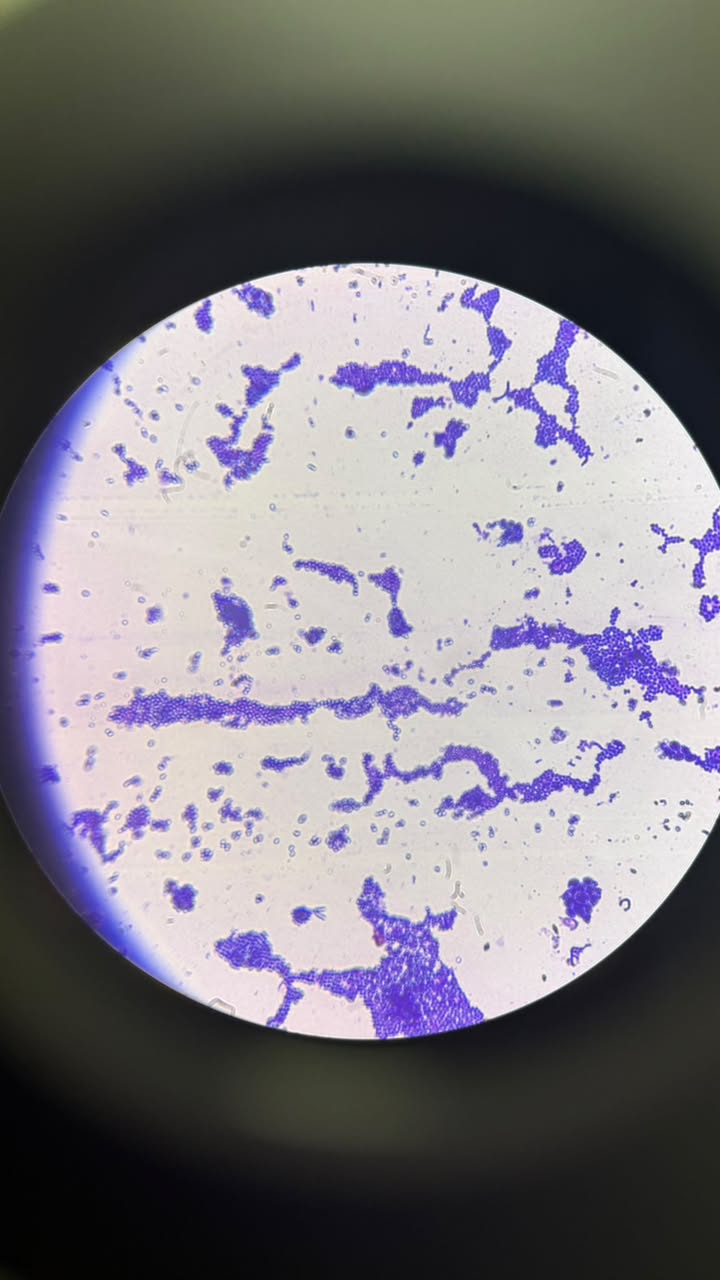

Supplement: Supplementary file 2 — Supplementary Material 2 [file 41598_2026_62193_MOESM2_ESM.jpeg]

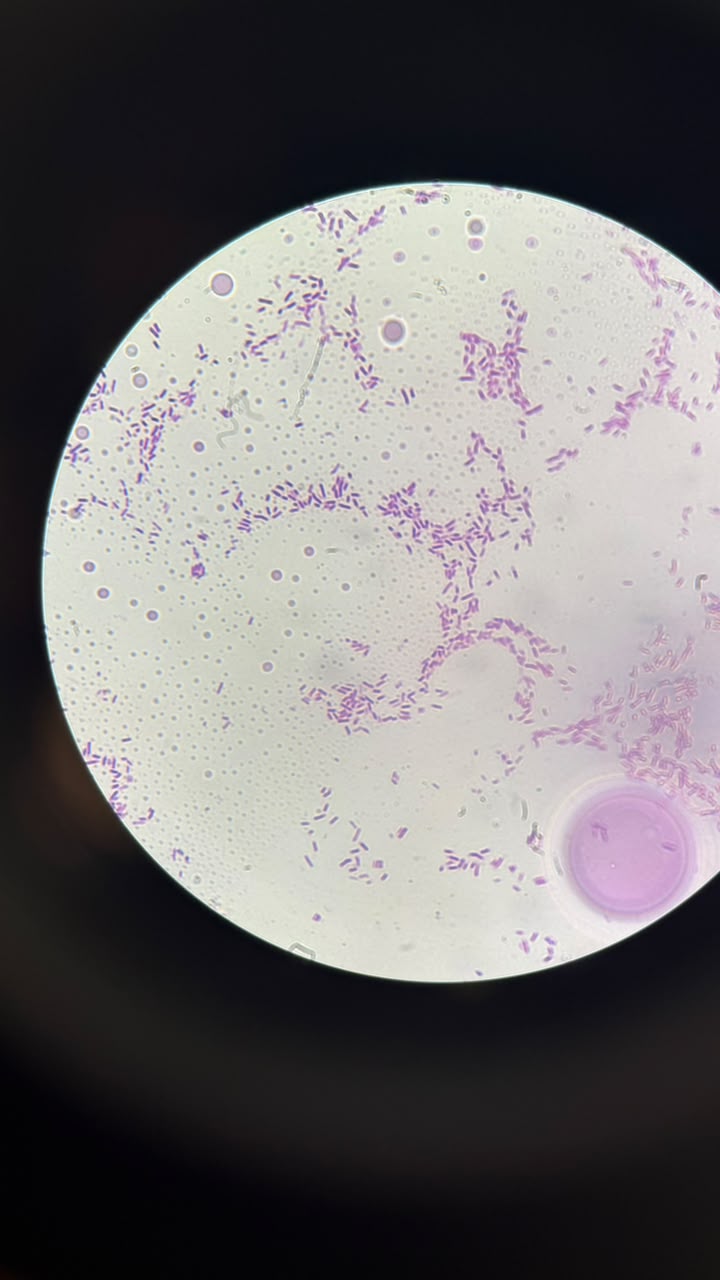

Supplement: Supplementary file 3 — Supplementary Material 3 [file 41598_2026_62193_MOESM3_ESM.jpeg]

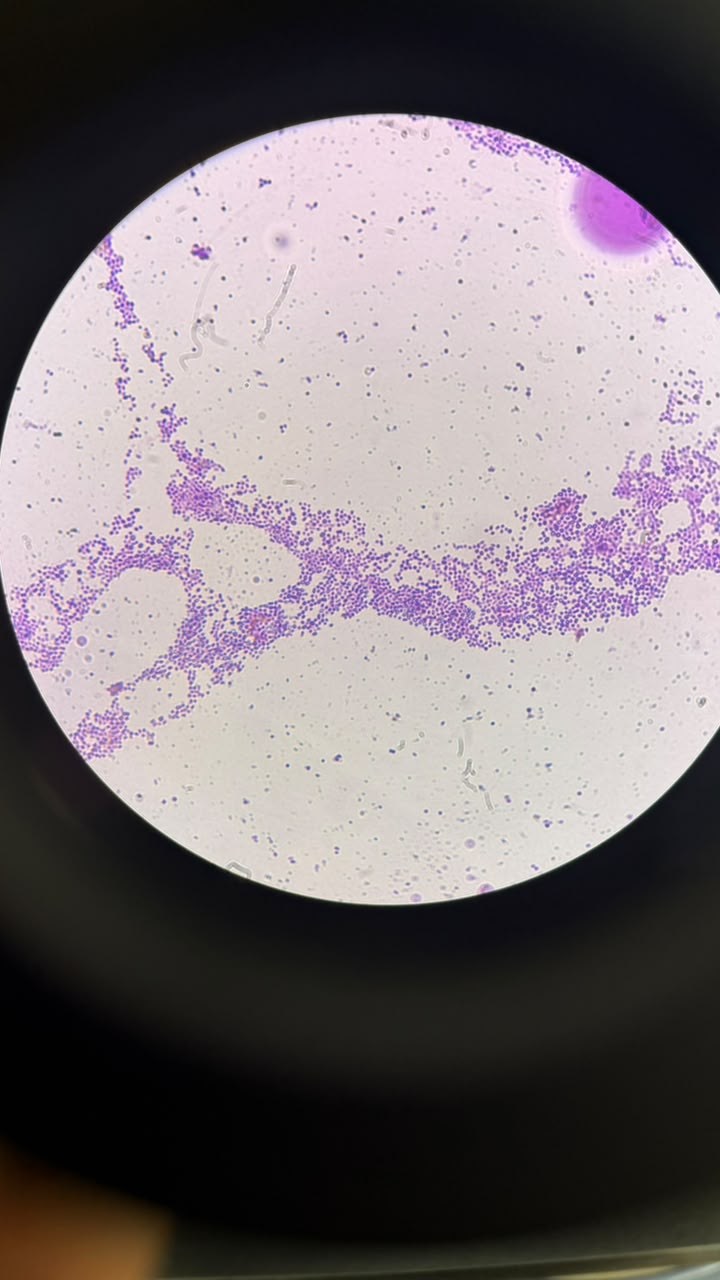

Supplement: Supplementary file 4 — Supplementary Material 4 [file 41598_2026_62193_MOESM4_ESM.jpeg]

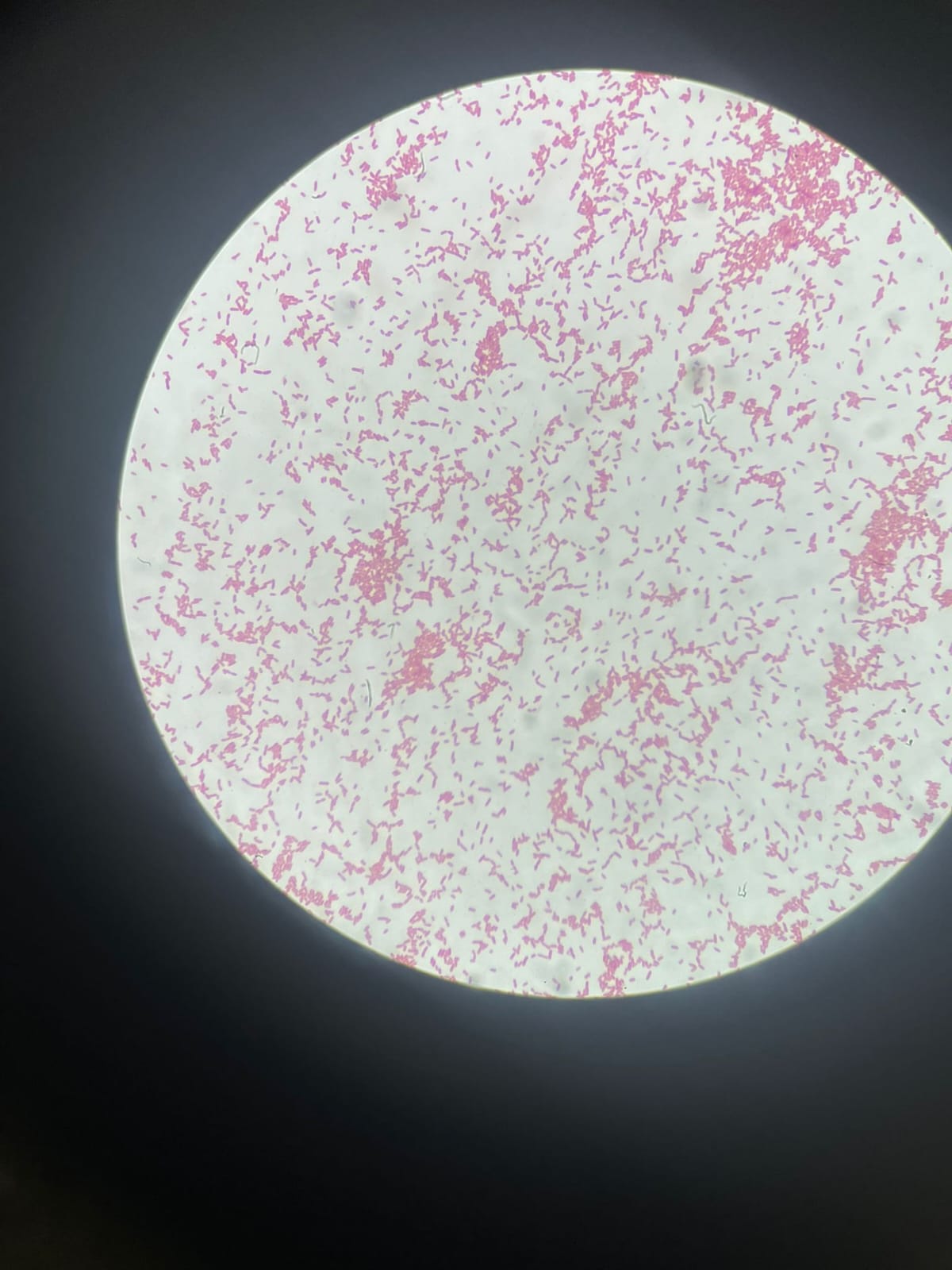

Supplement: Supplementary file 5 — Supplementary Material 5 [file 41598_2026_62193_MOESM5_ESM.jpeg]
